# Antimicrobial-Resistant *Staphylococcus* spp. Harbored by Hedgehogs (*Erinaceus europaeus*) in Central Italy

**DOI:** 10.3390/antibiotics14070725

**Published:** 2025-07-18

**Authors:** Fabrizio Bertelloni, Francesca Pauselli, Giulia Cagnoli, Roberto Biscontri, Renato Ceccherelli, Valentina Virginia Ebani

**Affiliations:** 1 Department of Veterinary Science, University of Pisa, Viale delle Piagge 2, 56124 Pisa, Italy; fabrizio.bertelloni@unipi.it (F.B.); f.pauselli@studenti.unipi.it (F.P.); giulia.cagnoli@phd.unipi.it (G.C.); r.biscontri@studenti.unipi.it (R.B.); 2 CRUMA-LIPU, Via delle Sorgenti 430, 57121 Livorno, Italy; apusvet.cruma@libero.it; 3 Centre for Climate Change Impact, University of Pisa, Via del Borghetto 80, 56124 Pisa, Italy

**Keywords:** antimicrobial resistance, *Erinaceus europaeus*, intestine, nasal swab, oral swab, *Staphylococcus* spp.

## Abstract

Background/Objectives: European hedgehogs (*Erinaceus europaeus*) are present in areas where there is human activity; therefore, they can be a source of pathogens for other animals and humans. Methods: Eighteen hedgehog carcasses were collected and analyzed for *Staphylococcus* spp. Isolated strains were typed and analyzed for exfoliative toxins genes and the phenotypic and genotypic characteristics of antimicrobial resistance. Results: A total of 54 strains were isolated and typed as *S. aureus*, *S. xylosus*, *S. sciuri*, *S. pseudintermedius*, *S. simulans*, *S. chromogenes*, *S. epidermidis*, *S. hyicus*, and *S. lentus*. No strains had the *eta* and *etb* genes coding for exfoliative toxins. Overall, 39/54 (72.20%) isolates showed phenotypic resistance to at least one antimicrobial and 21/54 (38.80%) showed more than one resistance. The lowest efficacy was observed for erythromycin, with 40/54 (74.08%) strains classified as intermediate and 6/54 (11.11%) classified as resistant. Among the 29 isolates shown to be penicillin-resistant, 11 (37.93%) were oxacillin-resistant, with a minimum inhibitory concentration (MIC). Among the 54 staphylococcal strains, 2 (3.70%) were resistant to vancomycin, both with an MIC value equal to the maximum concentration of the antibiotic tested (256 μg/mL) and 2 (3.70%) had an intermediate resistance profile with an 8 μg/mL MIC value. No strains had the genes *vanA* and *vanB*. Two of the 29 (6.90%) penicillin-resistant strains had the *blaZ* gene; 8 (27.13%) strains had the *mecA* gene. Overall, 2/54 (3.70%) isolates were classified as extensively drug-resistant (XDR) and 9/54 (16.66%) were classified as multidrug-resistant (MDR). Conclusions: Hedgehogs can harbor antimicrobial-resistant staphylococci and can be sources of these bacteria for other animals and humans. They can also serve as bioindicators of the pathogens and antimicrobial-resistant bacteria circulating in a given habitat.

## 1. Introduction

Human population growth, increased urban waste, and the destruction of natural habitats are leading wildlife to increasingly interact, both directly and indirectly, with humans [1]. Therefore, wild animals, particularly small mammals, such as hedgehogs, may have an important role in the epidemiology of zoonotic pathogens [2].

The western European hedgehog (*Erinaceus europaeus*) is a small, spiny mammal widespread throughout western Europe. Hedgehogs are increasingly present in areas with human activity, such as gardens in residential areas and rural villages [3], where they easily find food and water [4]. Hedgehogs are known to be sources of pathogens, such as *Leptospira* spp., *Salmonella* spp. and other *Enterobacteriaceae*, for humans and other animals [5]. Their role as a source of antimicrobial-resistant bacteria is particularly interesting from a One Health perspective. The inappropriate use of antibiotics in human and veterinary medicine, environmental contamination by antimicrobial residues, and ineffective infection control measures are responsible for the spread of antimicrobial-resistant bacteria among domestic and wild animals [6].

Some studies have focused on antimicrobial-resistant *Staphylococcus aureus* infection in hedgehogs. In 1964, Smith and Marples reported the first description of penicillin-resistant *S. aureus* in hedgehogs in New Zealand [7]. Subsequently, other investigations found methicillin-resistant *S. aureus* (MRSA) with variable prevalence rates. Similar prevalences (64% and 60%) were found in two different surveys in Sweden [8,9]; a similar result was obtained in Denmark (61%) [4]. Very low rates were detected in other investigations: only two (2/199) hedgehogs carrying *mecC*-MRSAs were detected in Sweden and two (2/199) in Germany [10,11]. In a study carried out in Austria, 40 different wild animal species were sampled, with 3 European brown hares, 1 European otter, and 1 hedgehog carrying *mecC*-MRSA [12]. A prevalence of 1% (2/200) of hedgehogs harboring MRSA was found in Hungary [13]. Recently, among 139 hedgehogs analyzed in France, 128 were *S. aureus* carriers and 25 (18%) presented with an MRSA isolate [14].

Conversely, data about the occurrence and characteristics of virulence and antimicrobial resistance of staphylococci belonging to other species in hedgehogs are scanty. In addition, to the best of our knowledge, surveys on staphylococcal infections in hedgehogs have not been conducted in Italy.

The aim of the present study was to verify the occurrence of *Staphylococcus* species in European hedgehogs living in central Italy and to analyze the isolates, evaluating their virulence genes, particularly those involved in the production of exfoliative toxins, and their phenotypic and genotypic characteristics of antimicrobial resistance.

## 2. Results

### 2.1. Animals

A total of 18 hedgehogs (9 males and 9 females) were included in the study. Three animals were classified as juvenile, with bodyweights ranging between 65.97 g to 92.13 g; the remaining 15 animals were adults with bodyweights ranging from 345 g to 750.68 g. Table 1 summarizes the morphometric data of the analyzed hedgehogs.

### 2.2. Bacteriological Analyses

#### 2.2.1. *Staphylococcus* Isolation

A total of 54 *Staphylococcus* strains were isolated from the 54 samples collected and analyzed. The strains were cultured from all samples except the intestine of H13; two strains were isolated from the intestine of H6. The isolates were confirmed to belong to the *Staphylococcus* genus by PCR. Among the 54 strains, 9 (16.6%) were identified as *S. aureus* by species-specific PCR. Forty-five strains were identified by API Staph ^®^ (bioMérieux SA, REF 20 500, Marcy l’Etoile, France) as: *S. xylosus* (16/54, 29.62%); *S. sciuri* (8/54, 14.81%); *S. pseudintermedius* (6/54, 11.11%); *S. simulans* (5/54, 9.25%); *S. chromogenes* (5/54, 9.25%); *S. epidermidis* (2/54, 3.70%); *S. hyicus* (2/54, 3.70%); and *S. lentus* (1/54, 1.85%). Overall, 17/54 (31.48%) isolates were coagulase-positive staphylococci (CoPS) of the following species: *S. aureus*; *S. pseudintermedius*; and *S. hyicus*. The remaining 37/54 (68.52%) were coagulase-negative staphylococci (CoNS) of the following species: *S. xylosus*; *S. sciuri*; *S. simulans*; *S. chromogenes*; *S. epidermidis*; and *S. lentus* (1/54, 1.85%). Both *S. aureus* and *S. hyicus* were isolated from the intestine of H6. In most cases, two or three staphylococcal species were found in each tested hedgehog, as summarized in Table 2.

No statistically significant differences emerged in the distribution of staphylococcal species in relation to animal sex and site of isolation (*p* > 0.05). *S. epidermidis* and *S. sciuri* were more frequently detected in juvenile than in adult animals (*p* < 0.05).

CoPS and CoNS were equally distributed in relation to sex, age, and site of isolation, without statistical differences (*p* > 0.05).

#### 2.2.2. Antimicrobial Sensitivity Tests

Disk Diffusion Method

The results of the disk diffusion test are reported in Table 3. Overall, 39/54 (72.20%) isolates showed phenotypic resistance to at least one antimicrobial and 21/54 (38.80%) showed more than one resistance. On the other hand, 15/54 (27.78%) isolates were resistant to none of the tested antimicrobials. Briefly, high percentages of susceptible isolates were detected to many antimicrobials: 96.30% to amikacin, 94.44% to trimethoprim–sulfamethoxazole, and 90.74% to gentamycin and amoxicillin–clavulanate. Even though no very high resistance rates were observed, significant percentages of intermediate strains were found. A high prevalence (29/54, 53.70%) of resistant isolates was found for penicillin. Erythromycin showed the lowest efficacy, with most of the strains (40/54, 74.08%) classified as intermediate and 6/54 (11.11%) as resistant. A high percentage (22/54, 40.74%) of intermediate strains was also observed for enrofloxacin; 5/54 (9.26%) isolates were resistant to this antimicrobial. A high percentage of resistant strains (18/54, 33.34%) was found for rifampicin. A relevant percentage (12/54, 22.22%) of intermediate isolates was found for ciprofloxacin.

*Staphylococcus* spp. strains isolated from oral swabs showed high levels of resistance to penicillin (11/18, 61.11%) and rifampicin (9/18, 50.00%) and many strains presented intermediate resistance to enrofloxacin (9/18, 50.00%) and erythromycin (15/18, 83.33%). Similarly, *Staphylococcus* spp. strains isolated from nasal swabs showed higher resistance to penicillin (10/18, 55.55%) and rifampicin (5/18, 27.77%) than average; a high percentage of intermediate strains was also observed for enrofloxacin and ciprofloxacin (6/18, 33.33%). The highest percentages of resistant strains, among those isolated from the intestines, were found for penicillin (8/18, 44.44%) and rifampicin (4/18, 22.22%); 66.66% (12/18), 38.88% (7/18), and 22.22% (4/18) of the isolates were intermediate for erythromycin, enrofloxacin, and ciprofloxacin, respectively.

No statistical differences (*p* > 0.05) emerged in relation to animal sex, age, and sample type (oral swab/nasal swab/intestine) when considering the resistances to the various antimicrobials tested.

Resistance to amoxicillin–clavulanate was higher in CoPS than CoNS (*p* < 0.05), and, similarly, intermediate resistance to erythromycin was higher in CoPS than CoNS (*p* < 0.05). Coagulase-positive and coagulase-negative staphylococci did not show other differences in the resistance/intermediate/susceptibility distribution in relation to the other antimicrobials evaluated (*p* > 0.05).

Finally, considering the species of staphylococci: *S. xylosus* was more resistant to penicillin than the other species (*p* < 0.05); *S. aureus* was more resistant to amoxicillin/clavulanate and cefoxitin than the other species (*p* < 0.05); *S. pseudintermedius* showed an intermediate result for enrofloxacin more often than other species (*p* < 0.05); *S. epidermidis* was more resistant to gentamicin and SXT than other species (*p* < 0.05). No statistical differences emerged in relation to the remaining antimicrobials and bacterial species (*p* > 0.05).

Minimum Inhibitory Concentration (MIC)

Among the 54 *Staphylococcus* spp. strains, 2 (3.70%) (H2A, H6A) were resistant to vancomycin, both with an MIC value equal to the maximum concentration of antibiotic tested (256 μg/mL). Two (3.70%) (H9C, H12B) had an intermediate resistance profile, with an 8 μg/mL MIC value. The remaining 50 (92.60%) isolates were found to be sensitive, with the following MIC values: ≤0.5 μg/mL (17/54, 31.48%); 1 μg/mL (10/54, 18.52%); 2 μg/mL (16/54, 29.60%); and 4 μg/mL (7/54, 12.96%).

Among the 29 *Staphylococcus* spp. isolates (11/18 from oral swabs, 10/18 from nasal swabs, and 8/18 from intestines) found to be resistant to penicillin by the disk diffusion method, 11 (37.93%) were oxacillin-resistant, with MIC values of 1 μg/mL for non-*S. aureus* strains (8/29, 27.58%), and values of 4 μg/mL (H8A), 8 μg/mL (H16A), and 16 μg/mL (H16B) for *S. aureus* isolates. The remaining strains (18/29, 62.07%) were found to be oxacillin-susceptible, with MIC values of 0.5 μg/mL (10/29, 34.48%), 0.25 μg/mL (4/29, 13.79%) and ≤0.125 (3/29, 10.34%) for non-*S. aureus* strains, while one *S. aureus* strain (H8B) had an 0.5 μg/mL MIC.

Resistance Profiles

Seventeen different antimicrobial resistance profiles were identified in the analyzed strains. In detail, 15 isolates did not show resistance to the tested antimicrobials, whereas the other isolates were resistant to between one and nine antimicrobials. Based on the resistance profiles, 2/54 strains (3.70%) were classified as extensively drug-resistant (XDR) and 9/54 (16.66%) were classified as multidrug-resistant (MDR), whereas 43/54 (79.62%) did not fall into any resistance class (Table 2). No strain was pandrug-resistant (PDR).

No statistical differences emerged in the distribution of multi-drug resistant (MDR + XDR) strains in relation to animal sex, age, sample type (oral swab/nasal swab/intestine), CoPS or CoNS, or bacterial species (*p* > 0.05).

### 2.3. Molecular Analyses

Out of the 29 penicillin-resistant *Staphylococcus* spp. strains, 2 (6.90%) had the *blaZ* gene, 1 *S. epidermidis* was isolated from a nasal swab (H1B) and 1 *S. epidermidis* was isolated from the intestine (H1C) of the same animal. Eight (27.13%) strains had the *mecA* gene: four from oral swabs (H1A, H2A, H14A, H17A); two from nasal swabs (H1B, H18B); and two from the intestines (H1C, H18C). No strain presented the *mecC* gene (Table 2). It was not possible to assign an SCC*mec* type to the *mecA*-positive strains; they showed the best homology with SCC*mec* type V or VII, being positive for the *ccr*C1 but negative for the *mec* class C2 or C1.

All vancomycin-resistant and intermediate strains were negative for the genes *vanA* and *vanB*. PCR carried out in all staphylococcal strains to detect the virulence genes *eta* and *etb* coding to produce the exfoliative toxins; no positive isolates were detected.

## 3. Discussion

The isolation of numerous strains of *Staphylococcus* belonging to distinct species indicates the large diffusion of these bacteria in the analyzed hedgehogs. The distribution of species varies considerably in relation to the anatomical sites, with some hedgehogs hosting the same staphylococcus at multiple sites (e.g., *S. epidermidis* in H1 and *S. intermedius* in H15) and others showing a higher diversity of species; however, no correlation between a given staphylococcal species and anatomical site of isolation emerged, even though the small number of tested animals was not statistically significant.

Overall, we found a predominance of coagulase-negative (CoNS) strains (72.22%), compared to coagulase-positive (CoPS) strains (27.77%). CoNS are usually considered commensal bacteria that can act as opportunistic pathogens. Among these, *S. xylosus* was the species most frequently encountered (29.62%); it is known as a commensal bacterium that generally occurs on the skin and mucous membranes of several animals. It is ubiquitous in nature, persisting in soils and sediments [15].

The other CoNS most frequently isolated were *S. sciuri* (14.81%), *S. simulans* (9.25%), and *S. chromogenes* (9.25%). *S. sciuri* has been commonly considered commensal in the oral cavity in rodent species and is sometimes associated with serious infections in humans [16,17,18,19]. Martel et al. found *S. sciuri,* as well as *S. aureus, S. simulans, S. xylosus, S. microti, S. fleurettii, S. pettenkoferi,* in association with *Corynebacterium ulcerans*, in deceased hedgehogs in Belgium [20]. *S. simulans* can cause several diseases in humans and animals; in addition, a report described a case of dermatosis characterized by broad, well-circumscribed hyperkeratosis and alopecia on the back of a household pygmy hedgehog (*Erinaceous albiventris*) [21]. *S. chromogenes* is recognized as one of the most frequent staphylococcal species causing subclinical intramammary infections in dairy cattle [22]; however, no data are available about its occurrence in hedgehogs.

Few strains of *S. epidermidis* and *S. lentus* were isolated; these CoNS are rarely associated with infections in humans and animals. *S. lentus* was previously isolated in Portugal from a hedgehog as the causative agent of pyometra [23].

Among CoPS, *S. aureus*, *S. pseudintermedius* and *S. hyicus* were cultured in 16.6%, 11.11%, and 3.70% of the analyzed samples, respectively. *S. aureus* has been found in hedgehogs in some previous investigations, whereas bacteria of the *Staphylococcus intermedius* group (SIG) have been related to infection in these animals living in urban areas of Finland only by Rautio et al. in 2016 [24]. The same authors reported the isolation of *S. hyicus*, the causative agent of exudative epidermitis of pigs [25], from sick hedgehogs.

Overall, 72.20% (39/54) of the strains isolated in this study showed phenotypic resistance to at least one antimicrobial and 38.80% (21/54) showed more than one resistance. The presence of XDR (3.70%) and MDR (16%) strains, and the variability of resistance profiles among the analyzed isolates, highlighted the diffusion in the antimicrobial resistance issue within the staphylococcal population. The nine MDR isolates belonged to distinct species, although *S. xylosus* was the most frequent, whereas both XDR strains were *S. epidermidis*. Most of these isolates were oxacillin-resistant, although not all had the investigated resistance genes. In particular, the two XDR strains had both *blaZ* and *mecA* genes but only one MDR had the gene *mecA*.

The relevant MIC values for vancomycin found in two strains (one *S. aureus* and one *S. sciuri*) and intermediate resistance values (one *S. pseudintermedius* and one *S. xylosus*) suggested the presence of strains potentially resistant to this antibiotic, which is considered as a last-line treatment for infections caused by resistant staphylococci. A thickening of the cell wall due to the overlapping of peptidoglycan has been reported in both *S. aureus* and CoNS resistant to glycopeptides [26]. Recent studies suggest that *S. aureus* may acquire genes for vancomycin resistance from enterococci [27]. However, vancomycin-resistant and intermediate strains tested in our study were negative for both *vanA* and *vanB* genes. Intermediate resistance to vancomycin in staphylococci, especially *S. aureus*, can also be due to the accumulation of multiple mutations in several chromosomal genes involved in peptidoglycan synthesis; the same non-transferable mechanism could also be related to the development of vancomycin resistance [28].

The frequent detection of *blaZ* and *mecA* genes in methicillin/oxacillin-resistant strains confirmed the involvement of these genes in this antimicrobial resistance. In particular, *mecA* was the most frequently found gene in isolates with an equal distribution between the oral, nasal, and intestinal sites. Conversely, the absence of the *mecC* gene in all staphylococcal strains indicates its minor relevance in the resistance of these bacteria. However, *mecC* has been previously found in MRSA strains isolated from hedgehogs in Hungary [13] and in Sweden [10]. Furthermore, one study found *mecC*-MRSA in river water in an area where *mecC*-MRSA had been isolated from wild boars and fallow deer, suggesting that water may be a shared site of exposure or transmission between various animal species and the environment [29].

Other studies supported the fact that carriage of the penicillin-producing dermatophyte *Trichophyton erinacei* in hedgehogs may impose a selective pressure that could facilitate the emergence of resistant variants of bacteria, such as *mecC*-MRSA, but also that the *blaZ* gene, which produces beta-lactamase and is in close proximity to the *mecC* gene on *mecXI* of SCC, could influence the selection of *mecC*-MRSA in hedgehogs [9].

Methicillin-resistant CoNS isolates are, in general, more often multi-resistant than isolates susceptible to this antimicrobial molecule [26]. However, the results of the present study showed that not all methicillin-resistant strains present beta-lactam resistance genes, indicating a phenotypic resistance, associated with alternative mechanisms for these strains by other authors, such as changes in bacterial membrane permeability; the presence of active efflux pumps; mutations in penicillin-binding proteins (PBPs) (i.e., target proteins that reduce the affinity of the drug without the need for the *mecA* gene); and adaptive mechanisms such as the ability to form biofilms and regulatory mechanisms related to the contact of bacteria with chemicals present in the environment, including antibiotics [30]. In addition, there are variants of beta-lactamases, other than those encoded by the gene *blaZ*, that may be less common or undetectable by standard genetic testing methods [30]. No *S. aureus* strains in this study presented beta-lactam resistance genes; therefore, the antibiotic resistance found in *S. aureus* strains does not appear to be mediated by the *blaZ* or *mecA* genes, highlighting the need for further investigations to understand the mechanisms of antibiotic resistance. Indeed, there are very few studies detecting the presence of MRSA strains in hedgehogs; less information is available on CoNS. However, the detection of *blaZ* and *mecA* genes in XDR and MDR *S. epidermidis, S. sciuri*, and *S. chromogenes* strains, is noteworthy. The alleles of *mecA* associated with resistance are located on mobile genetic elements known as staphylococcal cassette chromosome *mec* (SCC *mec*) elements and can be found in *S. aureus* as well as in other staphylococcal species such as *S. epidermidis* and *S. sciuri* [10]. Several SCC*mec* variants exist; nowadays, 15 different SCC*mec* types are recognized. Some of these are associated with *S. aureus* strains circulating and acquired within hospital settings, called hospital-associated MRSA (HA-MRSA), while other types are more often detected in community-associated MRSA (CA-MRSA)—*S. aureus* strains circulating and acquired outside hospitals. Recently, some SCC*mec* types seem to be particularly associated with strains circulating among farm animals; these are called livestock-associated MRSA (LA-MRSA) [31]. Isolates analyzed in this study were not typable in relation to the SCC*mec*. Most of these could be potentially assigned to type V, associated with HA-MRSA and CA-MRSA, or to type VII, which is not associable to a specific epidemiological niche [31]. The SCC*mec* typing method was set up to investigate *S. aureus mecA*-positive strains; it is possible that it is not applicable to CoNS in the same manner, or, more probably, that different genes circulate among coagulase-negative staphylococci, mainly among non-clinical strains. Other authors reported a high frequency of detection of not-typable strains among CoNS isolated from the environment [32,33]. Similar to our results, they found that most of the not-typeable strains showed the most similarity with type V SCC*mec* [32]. As proposed by some authors, whole-genome sequencing is probably the best method to classify these strains [34].

According to some studies, species, such as *S. sciuri*, taxonomically considered the most primitive among staphylococci found mainly in rodents and primitive mammals, frequently harbor *mecA* alleles, suggesting the role of this staphylococcal species as a possible ancestor of the methicillin-resistance determinant for MRSA strains. However, it seems that the presence of the *mecA* gene in this species is not associated with beta-lactam resistance, suggesting that *mecA* may be a native genetic element with a physiological function not yet identified in this staphylococcal species [35]. The *blaZ* gene has also been identified as a cause of penicillin resistance among coagulase-negative staphylococci (CoNS), as well as in porcine *S. hyicus*. Resistance gene transfer between CoNS and *S. aureus* has also been reported, indicating that CoNS may act as a reservoir of resistance genes for *S. aureus*. It is therefore possible that different staphylococcal species present in the same microenvironment may exchange *blaZ* genes if the appropriate bacterial factors occur [36].

The predominance of CoNS isolates in exhibiting multidrug and antiseptic resistance, as well as their biofilm-forming capacity, is strongly indicative of selection processes facilitated mainly by the excessive use of antibiotics [26]. A high prevalence of species, such as *S. sciuri, S. xylosus* and *S. chromogenes*, were isolated from the nasal swabs of wild boars in a study conducted in Spain [37]. Another study detected the presence of multidrug- and methicillin-resistant *S. sciuri* strains isolated from wild ungulates [38]. These data support the assertion that the spread of methicillin-resistant and multidrug-resistant CoNS has increased over the years, becoming a worrying threat for both human and veterinary medicine [39].

With the exclusion of β-lactams, several strains showed non-susceptibility to fluoroquinolones (both enrofloxacin 50.00% and ciprofloxacin 29.63%), erythromycin (85.19%) and rifampicin (48.15%).

Regarding fluoroquinolones specifically, recent attention has been paid to their environmental contamination [40]. Enrofloxacin, in particular, is extensively used in veterinary medicine and is excreted through the urine and feces of treated animals [41]. This excretion results in the release of enrofloxacin and its metabolites into the environment, where they can be readily absorbed by various organisms—including bacteria, plants, invertebrates, and wild animals. The environmental presence of fluoroquinolones may contribute to the development of antimicrobial resistance, which could explain the elevated resistance levels observed in hedgehogs, a species with a broad and human-interconnected habitat.

Generally, lower percentages of staphylococci non-susceptible to these antimicrobials were reported by other authors. In particular, Monecke and colleagues detected two *mecC*-positive *S. aureus* in two hedgehogs in Sweden; both isolates were susceptible to erythromycin and rifampicin [10]. In another investigation carried out in Sweden in 2017, the authors found 5/35 erythromycin-resistant isolates and no ciprofloxacin-resistant isolates in staphylococci isolated from hedgehogs [8]. In Hungary, Sahin-Tóth and collaborators isolated 13 *S. aureus* strains form hedgehogs. All the isolates showed intermediate resistance to ciprofloxacin and 8% showed intermediate resistance to erythromycin [13]. Recently, in a large survey carried out in France, 128 *S. aureus* strains isolated from hedgehogs were analyzed; all were susceptible to enrofloxacin and three were resistant to erythromycin [14]. Finally, all 11 MRSA strains isolated from hedgehogs in Finland were susceptible to erythromycin and rifampicin [42]. However, it is important to note that all the studies reported above focused only on *S. aureus* or MRSA strains—this could be a possible explanation for the dissimilarity to our results.

The absence of the virulence genes *eta* and *etb*, encoding exfoliative toxins, in all the analyzed strains suggested the limited pathogenic potential of our isolates, although further characterizations would be necessary. In fact, we isolated species, such as *S. aureus, S. hyicus,* and *S. chromogenes*, that are known to cause skin infections in humans and animals by producing exfoliative toxins [43,44]. A relatively new exfoliative toxin gene homologue named *etE* [45], previously called *etD2* [10], was detected in an MRSA isolated from one hedgehog in Hungary [13].

Hedgehogs have been proven to be potential source of pathogens; however, they can also be used as sentinels to monitor the spread of microorganisms in a given environment. In fact, hedgehogs often become accidental victims of human activity; their carcasses can serve as a valuable source of data for epidemiological studies [5]. These animals often live in urban and peri-urban areas, where they come into contact with the feces and urines of pets, mainly dogs and cats, as well as with urban waste; furthermore, hedgehogs are frequently present, in rural areas, in proximity to livestock. In all cases, they can contract different pathogens, including antimicrobial-resistant bacteria.

The present study has some limitations. First, the survey was carried out on a small number of animals; therefore, the results do not allow for correlations between staphylococcal data and animal parameters (age, sex, site) to be defined even though statistical analyses were carried out. The findings of our study confirmed that hedgehogs often harbor different staphylococcal species; further analyses on additional virulence and antimicrobial genes could better define the potential pathogenic role of these bacteria. We focused on *eta* and *etb* genes because they are responsible for the production of the principal exfoliative toxins implicated in human skin damage [46]. Veterinarians, wildlife center staff, or others who manipulate hedgehogs for treatments are at risk of being infected by the staphylococci producing these toxins.

The genes related to resistance to beta-lactams and vancomycin were investigated because currently, resistance to these antimicrobials represent the most significant concern in the treatment of staphylococcal infections. Penicillin resistance, and, in particular, methicillin resistance, is a global threat to public health. Vancomycin is one of the first-line drugs for the treatment of MRSA infections; however, staphylococcal isolates with complete resistance to vancomycin have emerged in recent years [47]. Even though these resistances are the most significant concern, studies to evaluate additional antimicrobial resistance are necessary to obtain a more exhaustive overview of this threat.

## 4. Materials and Methods

### 4.1. Sampling

From February to July 2024, 18 hedgehogs (*E. europaeus*), found dead due to trauma, were collected at a wildlife recovery center located in central Italy. Only carcasses in good conservation condition were collected and stored in freezers at −20 °C; they were thawed before the necropsies, during which gender and body measurements were noted and tissue samples were collected. In particular, the body length, the hindfoot length, and jaw length were recorded for age determination. Animals with a body length of <16 cm, a hindfoot length of less than 3.6 cm, and a jaw length lower than 4.5 cm were considered as juveniles (before first hibernation), while the others were considered as adults (after first hibernation) [48]. Different specimens were sampled for successive bacteriological analyses. In detail, oral and nasal swabs, and a portion of the intestine, were collected from each animal.

### 4.2. Staphylococcus spp. Isolation and Characterization

All samples were placed in brain heart infusion (BHI) broth (Thermo Fisher Diagnostics, Milan, Italy), to which 6.5% of NaCl was added, and incubated at 37 °C for 24 h. Each broth culture was streaked onto mannitol salt agar (MSA) (Thermo Fisher Diagnostics) and the plates were incubated at 37 °C for 24 h. One or two colonies were selected from each sample, in relation to mannitol fermentation, and sub-cultured on tryptic soy agar (TSA) (Thermo Fisher Diagnostics). The isolates were initially confirmed as *Staphylococcus* spp. with Gram staining and catalase testing. Successively, they were analyzed by polymerase chain reaction (PCR) to confirm the genus and to identify *S. aureus*, using primers (Table 4) and protocols previously described [49]. Isolates that were found not to belong to this species were analyzed using the kit API Staph ^®^ (bioMérieux SA, REF 20 500, Marcy l’Etoile, France). All typed isolates were cultured in brain heart infusion broth (BHI) (Thermo Fisher Diagnostics) and stored at −80 °C, with the addition of 20% glycerol as a cryoprotectant.

### 4.3. Antimicrobial Susceptibility Tests

All *Staphylococcus* spp. isolates were tested for antimicrobial susceptibility using the disk diffusion method, as reported by CLSI [50]. The following antimicrobial disks (Thermo Fisher Diagnostics) were employed: penicillin (10 U); amoxicillin–clavulanate (20/10 µg); cefoxitin (30 µg); ceftiofur (30 µg); chloramphenicol (30 µg); tetracycline (30 µg); enrofloxacin (5 µg); ciprofloxacin (5 µg); gentamicin (10 µg); amikacin (30 µg); trimethoprim–sulfamethoxazole (1.25/23.75 µg); erythromycin (15 µg); and rifampicin (5 µg). *S. aureus* ATCC 25923 and *S. aureus* ATCC 29213 were included as internal quality controls.

Additionally, for vancomycin resistance, MIC was evaluated with the broth microdilution method [51].

Staphylococcal strains that showed resistance or intermediate resistance to cefoxitin or penicillin by the disk diffusion assay were tested for methicillin resistance by assessing the MIC to oxacillin [51]. *Staphylococcus aureus* isolates showing an MIC ≥ 4 μg/mL were considered resistant, whereas *S. pseudintermedius* and CoNS isolates showing an MIC ≥ 1 μg/mL were classified as resistant [52].

All results were interpreted in accordance with CLSI guidelines [52,53] and EUCAST guidelines for Amikacin and Rifampicin [54].

The antimicrobial resistance profiles were evaluated to categorize the staphylococcal isolates as multidrug-resistant (MDR), extensively drug-resistant (XDR), and pandrug-resistant (PDR) [55].

### 4.4. Molecular Analyses

All *Staphylococcus* spp. strains were analyzed by a multiplex PCR to detect the genes *eta* and *etb* coding for the production of the exfoliative toxin [56].

*Staphylococcus* spp. strains found to be phenotypically non-susceptible to penicillin were analyzed by three uniplex PCRs to detect the genes *blaZ, mecA,* and *mecC* coding for resistance to beta-lactams [57,58,59]. The isolates testing positive for one *mec* gene were subjected to staphylococcal cassette chromosome *mec* (SCC*mec*) typing, as previously described [60].

Strains not susceptible to vancomycin were analyzed by the PCR method to detect the genes *vanA* and *vanB* [61].

For this purpose, DNA was extracted from overnight cultures of each isolate using the Quick-DNA Miniprep Plus Kit (Zymo Research, Irvine, CA, USA), according to the manufacturer’s instructions. DNA was promptly employed in PCR assays carried out in an automated thermal cycler (SimpliAmp™ Thermal Cycler, Applied Biosystems, Waltham, MA, USA). PCR reactions were assessed in a 25 µL final volume, containing 12.5 µL DreamTaq Hot Start Green Master Mix (Life Technologies Italia, Milan, Italy); 0.1 µM of each primer; 3 µL of extracted DNA; and ultrapure water to reach the final volume. Sterile distilled water was added as a negative control, whereas DNA extracted from previously characterized strains were employed as positive controls.

Each protocol had an initial denaturation at 95 °C for 5 min, followed by 40 cycles at 95 °C for 1 min, annealing (temperatures reported in Table 4) for 1 min, and 72 °C for 2 min; a final extension at 72 °C for 10 min completed the reaction. Primers and PCR conditions are summarized in Table 4. PCR products were run in 1.5% agarose gel at 100 V for 45 min, using 100 bp DNA Ladder Ready to Load (Solis BioDyne, Tartu, Estonia) as the DNA marker; the gel was stained with ethidium bromide and then observed under UV light.

**Table 4:** Primers and PCR conditions used for the molecular analyses of the *Staphylococcus* spp. isolates.

Target Gene	Primers	Oligonucleotide Sequence (5′–3′)	Annealing Temperature (°C)	Amplicon Size (bp)	Ref.
*16S* *rRNA*	Staph756F Staph750R	AACTCTGTTATTAGGGAAGAACA CCACCTTCCTCCGGTTTGTCACC	58	756	[49]
*nuc*	Nuc1 Nuc2	GCGATTGATGGTGATACGGTT AGCCAAGCCTTGACGAACTAAAGC	58	279	[49]
*eta*	etaF etaR	ATATCAACGTGAGGGCTCTAGTAC ATGCAGTCAGCTTCTTACTGCTA	52	1155	[56]
*etb*	etbF etbR	CACACATTACGGATAATGCAAG TCAACCGAATAGAGTGAACTTATCT	52	604	[56]
*bla*Z	bla-Z F bla-Z R	CAGTTCACATGCCAAAGAG TACACTCTTGGCGGTTTC	50	772	[57]
*mec*A	MecA147F MecA147R	GTGAAGATATACCAAGTGATT ATGCGCTATAGATTGAAAGGAT	50	147	[58]
*mec*C	mecLGA251F mecLGA251R	GCTCCTAATGCTAATGCA TAAGCAATAATGACTACC	50	304	[59]
*vanA*	vanAF vanAR	GGGAAAACGACAATTGC GTACAATGCGGCCGTTA	54	732	[61]
*vanB*	vanBF vanBR	ATGGGAAGCCGATAGTC GATTTCGTTCCTCGACC	54	635	[61]

### 4.5. Statistical Analysis

The chi-squared test was used to evaluate correlations among bacterial species, the anatomical site of isolation, sex, age, and antimicrobial resistance. A *p*-value < 0.05 was considered significant.

## 5. Conclusions

The hedgehog is a synanthropic wild species that can act as a possible source of pathogens, including antimicrobial-resistant bacteria, for humans and domestic animals. Furthermore, these animals, as well as other wild animals, can serve as bioindicators and sentinels of pathogens and antimicrobial-resistant bacteria circulating in a given habitat. Data in this study highlight the need to implement microbiological surveillance programs in wild and synanthropic animals to better monitor the evolution of antimicrobial resistance and consequently promote One Health policies. Such interventions must be based on the prudent use of antibiotics, in human and veterinary medicine, to limit the selection of resistant bacterial strains. Antimicrobial resistance must also be studied by identifying the genes coding for the various resistances, including the less frequent ones, since this genetic material can pass, more or less easily, from one bacterial strain to another.

In addition, the present findings confirmed wild animals as potential sources of zoonotic agents for wildlife rescue center staff. Anybody who intends to treat sick, injured, and/or orphaned wild animals may be at risk of infection. In particular, wildlife workers should be aware of this concern when manipulating live or dead hedgehogs; they should wear gloves made of sting-resistant material and avoid contamination from oral, respiratory, and conjunctival mucosae.

## Figures and Tables

**Table 1:** Morphometric data, age, and gender of the tested hedgehogs.

Hedgehogs	Sex	Age	Body Length (cm)	Hindfoot Length (cm)	Jaw Length (cm)	Body Weight (g)
H1	M	J	12	2.4	1.3	65.97
H2	F	A	16	3.6	2.5	377
H3	M	A	19	3.6	3.3	427.30
H4	F	J	13	2.7	1.7	92.13
H5	F	A	19	3.6	2.5	345.7
H6	F	A	23	4.3	4	734.77
H7	M	A	19.5	3.9	3.7	554.56
H8	M	A	22.5	4	3	425.74
H9	M	A	26	4	3.5	662.40
H10	M	A	24	4.3	2.5	571.24
H11	M	A	20	3.9	4	611.70
H12	F	A	22	4	4.3	574
H13	F	A	21	3.8	4.2	469
H14	F	A	23	4,5	3.8	540.50
H15	M	A	24	4.0	4.0	638
H16	F	A	20	3.6	3	750.68
H17	F	A	19.5	3.6	3	400
H18	M	J	12	2.5	2.4	87.27

Legend. M: male; F: female; J: juvenile; A: adult.

**Table 2:** Staphylococcal species, resistance profiles, and antimicrobials genes detected.

Samples	Staphylococcal Species	Resistance Profile	Resistance Class	Oxacillin MIC	*blaZ*	*mecA*	*mecC*	*vanA/* *vanB*
H1A	*S. sciuri*	P		1 *	−	+	−	NE
H1B	*S. epidermidis*	P FOX C ENR CN SXT E	XDR	1 *	+	+	−	NE
H1C	*S. epidermidis*	P AMC FOX C ENR CIP CN SXT E	XDR	1 *	+	+	−	NE
H2A	*S. sciuri*	P RD VA	MDR	1 *	−	+	−	−
H2B	*S. xylosus*	P E RD	MDR	0.25	−	−	−	NE
H2C	*S. xylosus*	E		NE	NE	NE	NE	NE
H3A	*S. xylosus*	P RD		0.5	−	−	−	NE
H3B	*S. xylosus*	P RD		0.25	−	−	−	NE
H3C	*S. sciuri*			NE	NE	NE	NE	NE
H4A	*S. xylosus*	P RD		0.25	−	−	−	NE
H4B	*S. sciuri*			NE	NE	NE	NE	NE
H4C	*S. sciuri*	P		0.5	−	−	−	NE
H5A	*S. sciuri*			NE	NE	NE	NE	NE
H5B	*S. lentus*	TE		NE	NE	NE	NE	NE
H5C	*S. xylosus*	P C TE ENR CIP	MDR	0.5	−	−	−	NE
H6A	*S. aureus*	VA		NE	NE	NE	NE	−
H6B	*S. aureus*			NE	NE	NE	NE	NE
H6C1	*S. aureus*	RD		NE	NE	NE	NE	NE
H6C2	*S. hyicus*			NE	NE	NE	NE	NE
H7A	*S. simulans*			NE	NE	NE	NE	NE
H7B	*S. simulans*			NE	NE	NE	NE	NE
H7C	*S. simulans*			NE	NE	NE	NE	NE
H8A	*S. aureus*	P AMC FOX EFT		4 *	−	−	−	NE
H8B	*S. aureus*	P AMC FOX EFT		0.5	−	−	−	NE
H8C	*S. simulans*			NE	NE	NE	NE	NE
H9A	*S. aureus*	RD		NE	NE	NE	NE	NE
H9B	*S. xylosus*	P		0.5	−	−	−	NE
H9C	*S. pseudintermedius*	P		0.5	−	−	−	−
H10A	*S. chromogenes*			NE	NE	NE	NE	NE
H10B	*S. chromogenes*	RD		NE	NE	NE	NE	NE
H10C	*S. chromogenes*			NE	NE	NE	NE	NE
H11A	*S. xylosus*	P RD		0.25	−	−	−	NE
H11B	*S. xylosus*			NE	NE	NE	NE	NE
H11C	*S. hyicus*			NE	NE	NE	NE	NE
H12A	*S. xylosus*	RD		NE	NE	NE	NE	NE
H12B	*S. xylosus*	P		0.5	−	−	−	−
H12C	*S. xylosus*	P E RD	MDR	0.5	−	−	−	NE
H13A	*S. xylosus*	P C TE ENR CIP E	MDR	≤0.125	−	−	−	NE
H13B	*S. xylosus*	P C TE ENR CIP	MDR	≤0.125	−	−	−	NE
H14A	*S. sciuri*	P TE		1 *	−	+	−	NE
H14B	*S. xylosus*	P RD		0.5	−	−	−	NE
H14C	*S. pseudintermedius*	P		0.5	−	−	−	NE
H15A	*S. pseudintermedius*	RD		NE	NE	NE	NE	NE
H15B	*S. pseudintermedius*	RD		NE	NE	NE	NE	NE
H15C	*S. pseudintermedius*	RD		NE	NE	NE	NE	NE
H16A	*S. aureus*	P AMC FOX EFT		8 *	−	−	−	NE
H16B	*S. aureus*	P AMC FOX EFT		16 *	−	−	−	NE
H16C	*S. xylosus*	P FOX EFT CN AK RD	MDR	0.5	−	−	−	NE
H17A	*S. chromogenes*	P FOX RD	MDR	1 *	−	+	−	NE
H17B	*S. aureus*			NE	NE	NE	NE	NE
H17C	*S. simulans*			NE	NE	NE	NE	NE
H18A	*S. pseudintermedius*	P FOX EFT AK RD	MDR	≤0.125	−	−		NE
H18B	*S. chromogenes*	P		1 *	−	+	−	NE
H18C	*S. sciuri*	P		1 *	−	+	−	NE

Legend. H: hedgehog; A: oral swab; B: nasal swab; C: intestine; P: penicillin, AMC: amoxicillin–clavulanate, FOX: cefoxitin, EFT: ceftiofur, C: chloramphenicol, TE: tetracycline, ENR: enrofloxacin, CIP: ciprofloxacin, CN: gentamicin, AK: amikacin, SXT: trimethoprim–sulfamethoxazole, E: erythromycin, RD: rifampicin; VA: vancomycin; MDR: multidrug- resistant; XDR: extensively drug-resistant; *: methicillin resistant; +: PCR positive; −: PCR negative; NE: not evaluated.

**Table 3:** Results of the disk diffusion test carried out on 54 *Staphylococcus* spp. isolates.

Antimicrobial Class	Molecule	Susceptible		Intermediate		Resistant		Not Susceptible	
		n. of Isolates	%	n. of Isolates	%	n. of Isolates	%	n. of Isolates	%
**penicillins**	P	25	46.30	0	0.00	29	53.70	29	53.70
	AMC	49	90.74	0	0.00	5	9.26	5	9.26
**cephalosporins**	FOX	45	83.33	0	0.00	9	16.67	9	16.67
	EFT	43	79.63	5	9.26	6	11.11	11	20.37
**phenicols**	C	42	77.78	7	12.96	5	9.26	12	22.22
**tetracyclines**	TE	44	81.48	5	9.26	5	9.26	10	18.52
**fluoroquinolones**	ENR	27	50.00	22	40.74	5	9.26	27	50.00
	CIP	38	70.37	12	22.22	4	7.41	16	29.63
**aminoglycosides**	CN	49	90.74	2	3.70	3	5.56	5	9.26
	AK	52	96.30	0	0.00	2	3.70	2	3.70
**sulfonamides**	SXT	51	94.44	1	1.86	2	3.70	3	5.56
**macrolides**	E	8	14.81	40	74.08	6	11.11	46	85.19
**ansamycin**	RD	28	51.85	8	14.81	18	33.34	26	48.15

Legend. P: penicillin, AMC: amoxicillin–clavulanate, FOX: cefoxitin, EFT: ceftiofur, C: chloramphenicol, TE: tetracycline, ENR: enrofloxacin, CIP: ciprofloxacin, CN: gentamicin, AK: amikacin, SXT: trimethoprim–sulfamethoxazole, E: erythromycin, RD: rifampicin.

## Data Availability

All data obtained during this study are reported in the present manuscript.
